# Formulation of teaching strategies for graduation internship based on the experiential learning styles of nursing undergraduates: a non-randomized controlled trial

**DOI:** 10.1186/s12909-022-03221-0

**Published:** 2022-03-07

**Authors:** Cong Li, Yu Yang, Yancui Jing

**Affiliations:** 1 Vascular Surgery Department/Thyroid Surgery Department of the First Affiliated Hospital of China Medical University, No.155, Nanjing Bei Street, 110001 Shenyang, People’s Republic of China; 2 China Medical University, No.77, Puhe Road, Shenyang, 110122 People’s Republic of China

**Keywords:** Learning styles, Nursing undergraduates, Internship, Holistic clinical competence

## Abstract

**Purpose:**

To formulate scientific and effective teaching strategies for the graduation internship of nursing undergraduates, in order to improve their holistic clinical competence.

**Method:**

A before-after self-controlled study with cluster sampling was performed on the 78 senior nursing undergraduates that underwent a graduation internship at the department. Students were required to fill in the Kolb’s Learning Style Questionnaire and Holistic Clinical Assessment Tool on the date of admission to assess their learning style characteristics and holistic clinical competence, according to which targeted teaching strategies for their graduation internship were formulated. When leaving the department, the students were required to fill in the Holistic Clinical Assessment Tool again to assess the changes in their learning skills after rotation.

**Results:**

In terms of learning methods, nursing students scored 23.87 ± 6.11, 29.57 ± 5.03, 37.85 ± 6.87, and 28.73 ± 6.70 in Concrete Experience, Reflective Observation, Abstract Conceptualisation, and Active Experimentation, respectively. When the learning styles were ranked by composition ratio, 46 students (58.9%) were assimilators, 18 (23.1%) were convergers, 9 (11.5%) were divergers, and 5 (6.4%) were accommodators. The holistic clinical competence of students after rotation was significantly improved compared to before rotation (*P* < 0.01).

**Conclusion:**

Clinical teaching strategies for graduation internship that are formulated according to the experiential learning style of nursing undergraduates can effectively improve their learning skills and holistic clinical competence.

## Preface

During their last year, nursing undergraduates leave the campus and are admitted to hospitals for a clinical internship, which is a critical stage during which students acquire multiple clinical abilities and prepare for independent work. Undergraduate nursing students are not only required to have a solid knowledge of diseases but also a sound holistic clinical competence. Yet, traditional teacher-centered teaching strategies cannot always contribute to the development of clinical skills required for a nursing internship [1].

## Introduction

The focus of medical teaching has gradually shifted to student-centered teaching, allowing students to participate in decision making, fortifying their capacity to lead, and remembering how it feels to learn [2]. Effective learning is based on a combination of different learning styles and learning situations [3]. Learning styles are factors that directly affect students’ learning process; the understanding of those factors allows teachers to develop appropriate teaching methods to improve students’ performance [4]. Kolb’s learning style theory, based on the comprehensive theory of learning and development, is currently the most widely-used learning model for students [5]. Kolb describes the process of experiential learning as a leaning cycle based on four stages: 1) Concrete Experience (CE); 2) Reflective Observation (RO); 3) Abstract Conceptualization (AC); 4) Active Experimentation (AE). Based on individual preference, four learning styles have been developed: (1) the diverging learning style, which combines concrete experience and reflective observation; (2) the converging learning style, that combines abstract conceptualization and active experimentation; (3) the accommodating learning style, which combines active experimentation and concrete experience; (4) the assimilating learning style, that combines reflective observation and abstract conceptualization [6]. Kolb encourages learners to discover their own learning style in the process of experiential learning and make up for the learning abilities they are weak in, so as to improve the flexibility of the four-stage transformation of experiential learning and help individuals to improve their learning efficiency. He also encourages teachers to investigate learners’ learning styles before designing corresponding curriculum plans [7].

Due to the complexity of the clinical learning environment, the current clinical competence of nursing students in a clinical internship is barely satisfactory [8–10]. Understanding the different learning styles of nursing students during clinical teaching and carefully considering when designing a clinical internship environment to adapt to the students’ various learning characteristics may be conducive to the improvement of the effectiveness of graduation internship [11–13]. In addition, the level of teachers in different clinical departments was uneven, and there was no unified standard for clinical teaching. Therefore, it is necessary to develop individualized clinical teaching strategies according to the learning styles of nursing interns and assess their holistic clinical competence. The aim of this study was to formulate scientific and effective teaching strategies for the graduation internship of nursing undergraduates in order to improve their holistic clinical competence.

## Materials and methods

### Design

A non-randomized pre-post study was used to verify the effectiveness of the teaching strategies for graduation internship based on the experiential learning styles of nursing undergraduates.

### Study subjects

All Grade-2014, Grade-2015, and Grade-2016 four-year full-time nursing undergraduates who underwent graduation internship at the Vascular Surgery Department/Thyroid Surgery Department of the First Hospital of China Medical University between July 2017 and December 2019 were recruited with cluster sampling. Students were selected according to the following inclusion criteria: 1) enrolled in the full-time undergraduate nursing education; 2) no record of failing or being downgraded in university; 3) not attending other clinical internship experiences during school. The exclusion criteria were: 1) unwilling to participate in the study; 2) with physical or psychological disorders and/or unable to communicate normally; 3) cannot ensure their full participation in the study. A total of 78 nursing undergraduates that underwent graduation internship were finally included in the study. The baseline data of all undergraduate interns are shown in Table 1.

**Table 1:** Baseline Information of Undergraduate Nursing Interns

	Item	Number (%)
Gender	Male	10 (12.8)
	Female	68 (87.2)
Expected year of graduation	2018	26 (33.3)
	2019	28 (35.9)
	2020	24 (30.8)
Average score	Excellent	5 (6.4)
	Good	16 (20.5)
	Moderate	25 (32.1)
	Fair	32 (41.0)
Number of rotated departments	0	21 (26.9)
	1	24 (30.8)
	2	18 (23.1)
	3	15 (19.2)
Age	22.36 ± 0.09 years old	

The study was reviewed and approved by the Ethics Committee of the First Hospital of China Medical University, and the students were informed to follow the principle of voluntariness. All data were kept confidential, and all subjects signed informed consent.

### Study tool

#### The Kolb Learning Styles Inventory 3.1 (KLSI,V3.1) [14]

The Kolb Learning Styles Inventory contains 12 items, 4 choices for each item, which indicate the four stages in the learning process, i.e., Concrete Experience (CE), Reflective Observation (RO), Abstract Conceptualization (AC) and Active Experimentation (AE). Students were asked to assign a score (1 to 4 points) according to the degree of his/her conformity; 4 points, she/he fully agree with sentence/statement; 3 points, she/he agrees with sentence/statement; 2 she/he barely agrees with sentence/statement; 1 she/he do not agree with sentence/statement. Each item must contain 1, 2, 3, and 4, with a total score of 10 and a total score for all items is 120. The preference and learning style (diverging, converging, accommodating or assimilating) was obtained after calculating the sum of the score in the Kolb learning style model. After calculating the CE, RO, AC, and AE scores, the AC-CE and AE-RO scores were calculated and projected in the corresponding area by referring to the type chart attached to the KLSI scale. The final learning style of a nursing intern was then defined. The Cronbach coefficients of CE, RO, AC, AE, AC-CE, and AE-RO of the Chinese version of KLSI(V3.1) respectively were 0.84, 0.78, 0.83, 0.73, 0.86 and 0.81. The retest reliability of the whole scale was 0.54.

#### Holistic clinical assessment tool (HCAT)

The holistic clinical assessment tool (HCAT) was developed by Singapore scholar Wu et al [15]. It covers the clinical capabilities of nursing undergraduates after the internship with reference to knowledge, skills, attitudes, values, and ethics. HCAT contains 36 items across four dimensions, i.e., “Professional, legal and ethical practice (10 items)”, “Clinical nursing (8 items)”, “Leadership and nursing management (13 items)” and “Specialty development (5 items)”. It adopts the 4 Point Likert Scale, with 1 to 4 points indicating “unqualified”, “qualified”, “skilled” and “excellent”, respectively. The total score ranges between 36 and 144 points, and the higher the direct total score of all items, the higher the holistic clinical competence. The scale has been tested and validated among nursing undergraduates in Singapore [15]. The Cronbach coefficient of the Chinese version of HCAT was 0.96, the retest reliability was 0.93, and the average content validity of the scale was 0.97.

### Research method

Senior nursing undergraduates were required to fill in the basic information questionnaire, Kolb’s Learning Style Questionnaire, and Holistic Clinical Assessment Tool on the date of admission to assess their learning style types and understand their basic clinical adaptability. After 4 weeks of internship, they were required to fill in the Holistic Clinical Assessment Tool again to assess the teaching effect.

Outstanding nurses with a bachelor’s degree or above, working for over 5 years and having the critical nursing qualification were selected as clinical internship instructors. Specific clinical internship objectives and detailed task lists of technical nursing operations and medical record management and teaching hours were formulated, as shown in Table 2. Different clinical internship instructors were assigned according to different learning styles of the students, with two fixed ones for each learning style. The instructors formulated corresponding clinical teaching strategies according to the learning style of their students and the task lists. Targeted one-to-one clinical internship teaching strategies were formulated according to the learning style of each nursing interns to the department: (1) clinical teaching design for accommodating nursing interns: accommodating nursing interns rely on others’ information when developing solutions, and preferring practical problems. Clinical instructors developed a clear teaching plan, defined a specific learning objective, defined the relationship between the objective and solving practical problems, divided weekly teaching knowledge based on experience, and asked questions. (2) Clinical teaching design for diverging nursing interns: diverging nursing interns like to observe, reflect and ask questions, tend to study in groups, prefer collecting information on their own, and are good at finding answers from experience and doing detailed works. In the teaching process, instructors assigned group tasks gave nursing interns sufficient time for discussion and exchange and allowed them to refer to and learn from others’ opinions. (3) Clinical teaching design for converging nursing interns: converging nursing interns believe that all answers come from actions, think after actions, are prone to blind impulse, have a strong practical ability, and are not good at communication. Therefore, patient clinical instructors were assigned for nursing interns to encourage them to think and draw their own conclusions step by step by asking questions and using the guidance strategy, and to increase the practice of clinical education and enhance the ability to communicate with patients. (4) Clinical teaching design for assimilating nursing interns: Assimilating nursing interns do not like the knowledge lacking background information and think only without putting knowledge into practice. Clinical instructors provided abundant materials and background information before lectures or presentations to allow students to conclude the main points and improve their ability of independent thinking and gave more examples to guide students to apply knowledge in practice and encouraged them to participate in practice, so as to improve their practical capability.

**Table 2:** Task List of Technical Nursing Operations and Medical Record Management and teaching hours

Time	Teaching content	Teaching method	Class hour(h)
First week	Getting familiar with the environment and HIS system	Teaching and demonstrating	2
	Writing of nursing records and log books, and execution and verification of the doctor’s advice	Teaching and demonstrating	4
	Bed making, morning and evening bed cleaning, and vital sign measurement	Teaching and demonstrating	4
	Introduction to the commonly-used drugs of the department, the oral administration method, and the dosing technology	Classroom demonstration and bedside instruction	4
	Oral care, perineal care, and aerosol delivery technology	Classroom demonstration and bedside instruction	4
	Replacing and maintenance of drainage devices	Presentation and group discussion	2
Second week	Nursing for patients with thyroid disease	Lecture, round and group discussion	4
	Indwelling gastric tube and gastrointestinal decompression technique	Classroom demonstration and bedside instruction	4
	Phlebotomy and blood glucose testing	Classroom demonstration and bedside instruction	4
	Closed intravenous infusion technique and intravenous injection	Classroom demonstration and bedside instruction	4
Third week	Nursing for patients with peripheral vascular disease	Lecture, round and group discussion	4
	Training on nursing techniques and writing of nursing records	Classroom demonstration and bedside instruction	4
	Intramuscular injection and intradermal injection	Classroom demonstration and bedside instruction	4
	Use of intravenous indwelling needles	Classroom demonstration and bedside instruction	2
	Central oxygen uptake and enema technique	Classroom demonstration and bedside instruction	4
Fourth week	Nursing for patients with common emergencies in general surgery	Lecture, round and group discussion	4
	Tracheotomy nursing, bedside ECG and ECG monitoring technique	Classroom demonstration and bedside instruction	4
	Infusion pump technique and injection pump technique	Classroom demonstration and bedside instruction	4
	Appraisal on nursing techniques, implementation of nursing procedures and writing of nursing records	Classroom demonstration and bedside instruction	4

On the date of admission, the head nurse informed the interns of the course arrangements, appraisal requirements, and procedures of the 4-week internship. The main procedures were as follows: the instructor developed a teaching plan, established a WeChat group, and distributed the printed teaching plan and the list of internship items formulated based on Kolb’s experiential learning theory (Table 2). During Week 1, the instructor familiarized all students with the environment of the department. Consequently, students were asked to complete nursing records, measure vital signs, provide oral care, and administer drugs under the supervision and guidance of the instructor. They learned from the instructor on how to approach patients and their families. In Week 2, more complex specialized nursing operations were added; contents related to medical record management, such as the surgical treatment of hyperthyroidism and clinical nursing rounds for different typical diseases, were introduced. Students were familiarized with the environment of the wards. The head nurse made clinical rounds once a day and teaching rounds once every 2 weeks. During weeks 3 and 4, more complex specialized nursing operations were introduced.

Before leaving the department, instructors examined the interns for their technical operations and comprehensive skills, checked the completion of every task in the teaching manual, and made timely remediation. In addition, they collected the examination papers from the interns and reported their examination results upon leaving, and adjusted the future teaching methods and contents according to the feedback of interns.

### Data collection

Data were collected by Y. Y. (Yu Yang) and a trained investigator using the KLSI Questionnaire and Holistic Clinical Assessment Tool on the date of student admission. After 4 weeks of internship, the Holistic Clinical Assessment Tool was distributed again to all students. The investigation was performed in a classroom; the questionnaires were collected 30 min after distribution. The questionnaires were checked one by one.

### Statistical analysis

SPSS 21.0 (Statistical Program for Social Sciences 21.0 software (SPSS, Inc., Chicago, IL, USA)) was used to make statistical descriptions and analyses of the data. The baseline information of the undergraduate nursing interns was described as percentages; the composition of their existing learning styles upon admission was described as composition ratios, and the age of a subject and his/her score in the learning process after admission were described as Mean ± SD. The holistic clinical competence of nursing interns of different demographic characteristics was compared with t-test and one-way ANOVA; changes in the clinical adaptability of nursing interns before and after the implementation of teaching strategies were compared using the independent t-test. A *P* < 0.05 was considered to be statistically significant. Multiple linear regression analysis was performed on the screened factors influencing clinical adaptability. The independent variables were taken in the result of the one-way ANOVA. The holistic clinical competence of nursing interns was taken as the dependent variable. The specific assignment of the independent was based on the specific score.

## Result

### Baseline information of undergraduate nursing interns participating in the study

A total of 78 students were included in the study. There were 10 males, 68 females, with an average age of 22.36 ± 0.09 years old (Table 1**).**

The scores of undergraduate nursing interns to the department in the learning process are shown in Table 3**.** In terms of learning methods, nursing students scored 23.87 ± 6.11, 29.57 ± 5.03, 37.85 ± 6.87, and 28.73 ± 6.70 in Concrete Experience, Reflective Observation, Abstract Conceptualisation, and Active Experimentation, respectively.

**Table 3:** Scores of Undergraduate Nursing Interns in the Learning Process (x ± s)^−^

Classification	Score
Concrete Experience	23.87 ± 6.11
Reflective Observation	29.57 ± 5.03
Abstract Conceptualization	37.85 ± 6.87
Active Experimentation	28.73 ± 6.70

Existing learning styles of undergraduate nursing interns to the department are shown in Table 4**.** When the learning styles were ranked by composition ratio, 46 students (58.9%) were assimilators, 18 (23.1%) were convergers, 9 (11.5%) were divergers, and 5 (6.4%) were accommodators. Compared with that before rotation, the holistic clinical competence of students after rotation was significantly improved (*P* < 0.01).

**Table 4:** Composition of Learning Styles of Undergraduate Nursing Interns (*N* = 78)

Learning style	Number (%)
Divergers	9 (11.5)
Convergers	18 (23.1)
Accommodators	5 (6.4)
Assimilators	46 (58.9)

### Factors influencing the holistic clinical competence of nursing interns

The one-way ANOVA analysis showed that holistic clinical competence scores were significantly different for the average score, a number of rotated departments, and a type of learning style (*P* < 0.05). At the same time, there was no difference in gender, age, and expected year of graduation (*P* > 0.05). The average score, number of rotated departments and type of learning style in the one-way ANOVA were taken as independent variables, and the holistic clinical competence of nursing interns was taken as the dependent variable to perform multiple linear regression analysis, with the screening criteria for regression variables being α in =0.05 and α out =0.10.

The results showed that the average score, the number of rotated departments, and the learning style were the main factors influencing the holistic clinical competence of nursing interns (Table 5).

**Table 5:** Regression Analysis of Holistic Clinical Competence of Undergraduate Nursing Interns

Independent variable	Partial regression coefficient	Standard error	Standard regression coefficient	t value	*P*-value
Average score	1.201	0.088	0.369	14.749	0.000
Number of rotated departments	0.516	0.023	0.196	7.486	0.000
Learning style	2.379	0.849	0.069	2.784	0.000

Changes in the holistic clinical competence of nursing interns before and after the implementation of teaching strategies are shown in Table 6**.**

**Table 6:** Changes of Holistic Clinical Competence of Undergraduate Nursing Interns Before and After Internship at the Department (*N* = 78)

Dimension	Number of items	Before internship (x ± s)^−^	After internship (x ± s)^−^	t value	P-value
Professional, legal and ethical nursing practice	10	3.59 ± 0.41	4.16 ± 0.56	5.137	< 0.001
Management of care	8	3.21 ± 0.26	3.68 ± 0.47	5.580	< 0.001
Leadership and nursing management	13	2.89 ± 0.37	3.46 ± 0.66	4.768	< 0.001
Professional development	5	3.04 ± 0.51	3.45 ± 0.52	3.553	0.001
Total score	36	114.35 ± 13.54	133.23 ± 20.54	3.817	< 0.001

## Discussion

In the present study, we found that assimilators (58.9%) were in the majority, and 5 accommodators interns (6.4%) were in the minority. Interns were more likely to learn with the logic analysis method based on observation and conceptualization and lacked the skill to perceive information through concrete experience and processing information through active experimentation. The study results were consistent with Samira Hassanzadeh et al and Int J Health Sci (Qassim) et al [16–19], which found that the majority of undergraduate nursing students were assimilators, while few of them were accommodators. Yet, these data were inconsistent with Fatemeh et al [12, 10] which foudings were the most dominant learning style of the nursing students was the divergent one, which may be related to traditional education in China, where students have clear learning objectives and like to think and analyze, but are not good at practice. After an in-depth understanding of the individual differences and personalities of nursing interns, clinical instructors implemented personalized clinical teaching strategies matching their preferred learning styles so as to allow students to find their own “excitement” as soon as possible, thus arousing their enthusiasm and improving their performance [20].

Among all dimensions, students obtained the highest score in professional, legal, and ethical practice (3.59 ± 0.41) and the lowest score in leadership and nursing management (2.89 ± 0.37) before the intervention, which implied that the leadership and nursing management should be strengthened during clinical internship [21, 22]. After the implementation of personalized teaching strategies for an internship, the nursing professional, legal and ethical practice, clinical nursing, leadership, and nursing management, as well as the specialty development and holistic clinical competence, significantly improved. In this study, we should take students as the center in the process of clinical practice teaching, and give full consideration to learning style of students. It could ensure the learning interest of nursing student, and improve the effect of receiving knowledge. In addition, in clinical practice, we should pay attention to the formulation of simple and progressive learning task list, so that students could more easily accept knowledge, but also ensure teaching tasks would not cause teaching content deviation due to different clinical teachers. In future studies, it is suggested to expand the sample size and select students of the same grade to conduct investigations in the whole hospital.

## Conclusion

Teaching strategies for graduation internship developed based on Kolb’s experiential learning theory and students’ learning style characteristics may comprehensively improve the holistic clinical competence of undergraduate nursing interns.

### Study limitations

In this study, a non-random allocation of samples was performed. Also, the sample size was small. A true random sampling was not possible to perform due to educational and institutional limitations, given that researchers performed self-historical control before and after the intervention.

## Data Availability

All data generated or analysed during this study are included in this published article.

## Abbreviations

CE: Concrete Experience
RO: Reflective Observation
AC: Abstract Conceptualization
AE: Active Experimentation
ANOVA: Analysis of variance

## References

[1] van Wyngaarden A, Leech R, Coetzee I (2019). Challenges nurse educators experience with development of student nurses’ clinical reasoning skills. Nurse Educ Pract.

[2] Yousafzai YM, Baseer N, Fatima S, Ali A (2018). Changes in learning style preferences of postgraduates after entering a new learning environment. J Ayub Med Coll Abbottabad.

[3] Suliman WA (2010). The relationship between learning styles, emotional social intelligence, and academic success of undergraduate nursing students. J Nurs Res.

[4] Vizeshfar F, Torabizadeh C (2018). The effect of teaching based on dominant learning style on nursing students’ academic achievement. Nurse Educ Pract.

[5] Stander J, Grimmer K, Brink Y (2019). Learning styles of physiotherapists: a systematic scoping review. BMC Med Educ.

[6] Kolb AY, Kolb DA (2013). The Kolb learning style inventory 4.0: guide to theory, psychometrics. Res Appl Book.

[7] Kolb AY, Kolb DA (2017). Experiential learning theory as a guide for experiential educators in higher education. Exp Learn Teach Higher Educ.

[8] Joolaee S, Amiri SRJ, Farahani MA (2015). Iranian nursing students’ preparedness for clinical training: a qualitative study. Nurse Educ Today.

[9] Kenny P, Reeve R, Hall J (2016). Satisfaction with nursing education, job satisfaction, and work intentions of new graduate nurses. Nurse Educ Today.

[10] Ab Latif R, Mat Nor MZ (2019). Stressors and coping strategies during clinical practice among diploma nursing students. Malays J Med Sci.

[11] Brunt BA, Kopp DJ (2007). Impact of preceptor and orientee learning styles on satisfaction. J Nurs Staff Dev.

[12] Fatemeh SHIRAZI, Shiva HEIDARI (2019). The relationship between critical thinking skills and learning styles and academic achievement of nursing students. J Nurs Res.

[13] Çelik Y, Ceylantekin Y, Kiliç İ (2017). The evaluation of simulation market in nursing education and the determination of learning style of students. Int J Health Sci.

[14] Kolb DA (2015). Experiential learning: experience as the source of learning and development.

[15] Wu XV, Enskar K, Pua LH (2016). Development and psychometric testing of holistic clinical assessment tool (HCAT) for undergraduate nursing students. BMC Med Educ.

[16] Hassanzadeh S, Moonaghi HK, Derakhshan A (2019). Preferred learning styles among ophthalmology residents: an Iranian sample. J Ophthalmic Vis Res.

[17] Çelik Y, Ceylantekin Y, Kiliç İ (2017). The evaluation of simulation market in nursing education and the determination of learning style of students. Int J Health Sci (Qassim).

[18] EI-Gilany A, Abusaad FES (2013). Self-directed learning readiness and learning styles among Saudi undergraduate nursing students. Nurs Educ Today.

[19] Fogg L, Carlson-Sabelli I, C'arlson K (2013). The perceived benefits of a virtual community; effects of learning style, race, ethnicity, and frequency of use on nursing students. Nurs Educ Perspect.

[20] Shirazi F, Heidari S (2019). The relationship between critical thinking skills and learning styles and academic achievement of nursing students. J Nurs Res.

[21] Mianda S, Voce A (2018). Developing and evaluating clinical leadership interventions for frontline healthcare providers: a review of the literature. BMC Health Serv Res.

[22] van Diggele C, Burgess A, Roberts C, Mellis C (2020). Leadership in healthcare education. BMC Med Educ.

